# "The Hospital" Medical Book Supplement.—No. XIV

**Published:** 1909-01-16

**Authors:** 


### "THE HOSPITAL" MEDICAL BOOK SUPPLEMENT.—No. XIV.

CONTENTS.

**Medicine—**
The Diagnosis of Smallpox ... ... ... ... ... ... ... 411
The Medical Inspection of School Children ... ... ... 411

**Therapeutics—**
Physiological Principles in Treatment ... ... ... ... ... 412
A Dictionary of Medical Treatment: For Students and Junior Practitioners ... ... ... ... ... ... ... ... ... 412

**Clinical Pathology—**
Clinical Diagnosis ... ... ... ... ... ... ... ... 413
Physiology and Pathology of the Urine ... ... ... ... 413

**Nursing and Allied Subjects—**
Practical Gynæcology: a Manual for Nurses and Students ... 412
The Home Nursing of Sick Babies ... ... ... ... ... ... 412
Handbook for Attendants on the Insane ... ... ... ... 413
Infections and Parasitic Diseases ... ... ... ... ... 413

**Miscellaneous Items—**
Hints to Ships' Surgeons ... ... ... ... ... ... ... 414
First Lines in Dispensing ... ... ... ... ... ... ... 414
From an Easy Chair ... ... ... ... ... ... ... ... 414
Vital Economy: or, How to Conserve your Strength ... ... 414
Who's Who ... ... ... ... ... ... ... ... ... ... 414

### MEDICINE.

The Diagnosis of Smallpox. By T. F. Ricketts, M.D., M.R.C.P., Medical Superintendent of the Smallpox Hospitals of the Metropolitan Asylums Board. With 122 plates from photographs by J. B. Byles, M.B., F.R.C.S., Senior Assistant Medical Officer at the Smallpox Hospitals of the Board. (London: Cassell and Co., Ltd. Price 21s.)

Very seldom indeed does it fall to a reviewer to express an opinion on a book of such sterling worth and sound scientific spirit as this monograph by Dr. Ricketts and Mr. Byles. It is also some pleasure to entertain the conviction, which a perusal of its pages is well calculated to produce, that British medicine will derive an added reputation in whatever country this book circulates. The author lays great stress on the distribution of the focal rash of smallpox as the most important element in the diagnosis of the disease, as opposed to the anatomical characters of the individual lesions: the much greater frequency of modified smallpox, compared with pre-vaccination days, invalidates largely the distinctions based upon the consideration of the individual lesion upon which reliance was formerly placed in the differential diagnosis. To this proposition the first four chapters are devoted, and they are illustrated by a profusion of magnificent full-page photographs by Mr. Byles, illustrating the points under discussion. After that the individual lesion is discussed, and then the varieties of eruption and the eruptive fever. Here again excellent plates are freely used to illustrate the descriptions; some are stereoscopic, and others are examples of colour-photography by the Sanger-Shepherd process. The latter are extremely successful; they show the actual shades of colour with a fidelity which must surpass that of the most skilful artist, and are, indeed, object lessons of what we may expect in the medical text-books of the future. The other plates are naturally somewhat over-shadowed by these beautiful colour-photographs, but are really most admirable work, upon which Mr. Byles is to be highly complimented. Unluckily the copy before us lacks altogether the first four pages of chapter six, whereas the next four are printed twice over.

After these chapters the author passes on to the consideration of modified smallpox and of the secondary characteristics of the eruptions, and thence to septic and toxæmic conditions and their differential diagnosis. Especially valuable also are the chapters on the diseases with which confusion is likely to arise. As elsewhere, the illustrations of these diseases are of the highest class. The work concludes with the consideration of vaccinia, the use of vaccination in diagnosis, and second attacks. The letterpress is throughout scholarly and informed by a thoroughly scientific spirit; both design and execution leave little to be desired. We would commend an examination of this work not alone to the medical profession, but also to the opponents of vaccination. It is, of course, not in the least conceived for their instruction or admonition; but possibly the witness it bears may carry all the more conviction by reason of that very fact. The mere perception that an authority on smallpox so pre-eminently in a position to judge, and so admirably qualified to take advantage of his opportunities as Dr. Ricketts obviously is, does not think it worth while to argue about the protective effects of vaccination, but states his views as to just how much and just how little that protection amounts to, with a certainty as dogmatic as that with which he treats of, say, the existence of smallpox itself, should surely be a matter for reflection to the most fervent and fervid of the dissentients. It is hardly reasonable to expect them to expend a guinea for this purpose, but we are at liberty to point out that medical libraries are numerous and flourishing; and we cannot imagine any such institution which will not deem it necessary to subscribe for a copy of this book.

The Medical Inspection of School Children. A Series of Lectures delivered at the West London Post-Graduate College. (London: "The Medical Officer." Pp. 62. Price 1s. net.)

It is certainly a happy thought on somebody's part to reprint in collected form the six lectures on various aspects of school medical inspection which compose this pamphlet. The Education Administrative Provisions Act of 1907, which gives power to Local Education Authorities to make arrangements for attention to the health and physical condition of the children in elementary schools, has been taken advantage of by a very great number of Authorities, and in consequence many practitioners all over the country are now engaged in this branch of work. In the absence of any text-book especially designed to assist medical officers appointed under this Act, a symposium of members of a hospital out-patient staff is undoubtedly a good way of filling the gap. In the volume under notice are two lectures on the general scheme of inspection and on general diseases by Dr. Arthur Saunders, and one each on skin diseases by Dr. Abraham, eye diseases by Mr. Kenneth Scott, ear, nose, and throat troubles by Dr. H. J. Davis, and dental surgery by Mr. H. L. Williams respectively. Taken all round, the teaching contained in the lectures is sound and adequate, and those who have already undertaken or are about to undertake this kind of work will find the modest price of the publication an investment which they will certainly not regret.

### THERAPEUTICS.

Physiological Principles in Treatment. By W. Langdon Brown, M.D., F.R.C.P., Physician to the Metropolitan Hospital, Medical Registrar and Demonstrator of Physiology, St. Bartholomew's Hospital. (London: Baillière, Tindall, and Cox. 1908. Ppp. vii+344. Price 5s. net.)

It is by applying the principles of physiology that advances upon strictly scientific, as distinct from empirical, lines of treatment are to be expected. The whole difficulty lies in the fact that physiologists are far too apt to state as absolute facts things which, though apparently true at the time, soon require modification or even complete reversal in the light of fresh work. If one could be sure of the teachings of physiology, then the lines of treatment indicated by them would be safe to follow. As it is, however, the physiological teachings of 1898 may have indicated some method of treatment which those of 1908 indicate as absolutely detrimental. This is the weak feature of any work bearing the above title, and unless it were written by a level-headed and rather sceptical author it might do a good deal of harm. We think that Dr. Langdon Brown quite feels this, for he tells us that "the physiologist is still regarded a little suspiciously at the bedside. Perhaps he is in part himself to blame for that, for he is sometimes inclined to forget that observations made in the laboratory are not infallible, and are not necessarily more correct than clinical evidence. When I reflect that I am now teaching the exact opposite to many of the views held ten years ago, I feel that physiology can only come to the aid of medicine with becoming modesty and without overweening dogmatism." The author has the advantage of being both a clinician and a physiologist; he cannot be certain that his views upon physiology will be the same ten years hence as they are now, but he has done his best to correlate physiology and therapeutics in a helpful way. Opinions must necessarily differ on certain points, but Dr. Langdon Brown is always interesting, and he suggests a large number of helpful ideas.

A Dictionary of Medical Treatment: For Students and Junior Practitioners. By Arthur Latham, M.D., F.R.C.P., Physician to St. George's Hospital, etc. (London: J. and A. Churchill. 1908. Pp. 325. Price 6s. 6d. net.)

This modest volume, barely an inch thick, is full of the most clear, rational, and level-headed directions as to the treatment of all manner of medical maladies. It avoids panegyrics upon the one hand, and upon the other it gives good advice as to measures that may be taken to alleviate incurable conditions whose treatment is so often passed over in silence by the average text-book. There are, naturally enough, a considerable number of points upon which we do not agree with the author, but these scarcely detract from the great usefulness of the book as a whole. For instance, when we look up "Osteoarthritis" we find "*see* Rheumatoid Arthritis"; and when we turn to the latter we find no mention of the acute rheumatoid arthritis of children and of adult women. Dr. Latham may say that he does not agree with our nomenclature, but we would answer that his book, to be perfect, must make allowances for differences in names and should therefore subdivide the treatment of rheumatoid arthritis more than it has done. Apart from a few such points as this, however, we think the work admirable. It will be a boon to students, and probably to established practitioners also. It would perhaps have been still more useful if prescriptions had been written out in full.

### NURSING AND ALLIED SUBJECTS.

Practical Gynæcology; a Manual for Nurses and Students. By Netta Stewart and James Young, M.B., F.R.C.S.(E.). (Edinburgh: Oliver and Boyd. 1909. Price 5s. net.)

This is the second edition, much enlarged and extended, of the work entitled "Gynæcological Nursing," of which Miss Stewart was the sole author. The scope of the treatise is now extended to include everything except the diagnosis and treatment of actual diseases. Methods of examination, instruments and how to use them, antiseptic and aseptic preparations, after-treatment of all kinds of operations, complications, and, in fact, all that a gynæcologist and the sister of a gynæcological ward ought to know on these and kindred topics are dealt with in detail. Much of the instruction is necessarily elementary, but the subjects are discussed so thoroughly that we venture to assert that there are few who will not find something that is novel to them somewhere. Designed as the book is for students and nurses, the tone is quite rightly dogmatic, and the methods described are presumably those which prevail in Edinburgh. The iodine method of preparation of the skin for laparotomy, which has been recently recommended abroad by various surgeons, notably by Grossich, is one which we confess we have not seen used in London: it may be conceded that "iodine has a strong antiseptic influence," but we are a little sceptical about "the virtue which it possesses of attracting the white blood corpuscles" and so of equipping "the skin for resisting organismal infection." There are one or two minor details of the like kind in which practice over the border evidently differs from that in England: especially is this the case with the principles which dominate the after-treatment of laparotomies in respect to feeding and intestinal evacuation. The *régime* advised as to food and drink appears somewhat unnecessarily severe: great stress is laid upon anæsthetic vomiting, which (apart from that which is so frequent before consciousness is recovered) ought to be the exception rather than the rule. The administration of an aperient is deferred until the evening of the third or morning of the fourth day, and in certain cases even two or three days more. Another point whereon the authors differ from what is regarded as sound practice in England is in the use of the flatus tube, which is mentioned as a kind of last resort after enemas of magnesium sulphate and turpentine have failed to produce the desired result of relieving abdominal distension; whereas here it is regarded rather as a useful prophylactic and is ordered four-hourly as a routine. The book as a whole is one well worthy of close study by those to whom it is addressed.

The Home Nursing of Sick Babies. By Theresa M. Brewster. (London: The Scientific Press. Price 6d. net.)

In this unpretentious pamphlet are set forth a number of very highly practical hints to those who may have to undertake the nursing of a sick baby. These hints display an insight into the psychology of childhood which only an experienced and sympathetic nurse could attain: they do not attempt to give any technical instruction in nursing, but as reminders to hospital-trained women of some of the most striking differences between ward work and private nursing they supply just that for want of which so many nurses fail on first undertaking the latter. To all such this publication can be most strongly recommended.

Handbook for Attendants on the Insane. (Baillière, Tindall, and Cox. Crown 8vo. Price 2s. 6d. net.)

This new edition of the "Handbook for Nurses and Attendants on the Insane," published by authority of the Medico-Psychological Association, has been expected for a long time, and we are glad to see it appear. It is in every way a great improvement on the last edition, and is about double the size, containing a large amount of new matter. There has very rightly been added amongst this new matter a section on "The Mind in Health," which was badly needed in the former edition. The whole book has been carefully prepared, and though it has been found necessary to condense some subjects into a very small space, it is, on the whole, clear throughout. Some of the more advanced matter is printed in smaller type that it may be omitted by junior nurses. There are not many diagrams, and all except one are good, those of brains in health and disease being excellent. The diagram on page 193 of the motor and sensory tracts in the spinal cord, medulla, and brain will, we are sure, not be understood without much explanation. Had the motor and sensory fibres been printed in different colours it would have been plainer, but even then the crossed fibres suddenly ending, to restart on the opposite side, would not have made for lucidity. The paper and type are good, and the index appears to be full. The Medico-Psychological Association is to be congratulated on its labours.

Infections and Parasitic Diseases. By Millard Langfield, A.B., M.B. Pp. xvi+260, with 33 illustrations. (London: Messrs. Rebman. 1908. Price 5s. 6d. net.)

This book was primarily written for the use of nurses, and later rearranged to some extent in order that it should appeal to physicians and students also. It is a pity that any such rearrangement was attempted. Had comparative simplicity been maintained throughout, had the volume been constructed simply and solely for nurses, it is probable that it would have been of greater service to medical men than it is in its present form. Just as the letterpress is of uneven quality and style, so is the subject-matter. An out-of-the-way malady like dum-dum fever is described, and yet there is practically no discussion of a matter of such every-day importance as the relation of milk to tuberculosis; the nurse or student will find the chief points about madura foot and craw-craw mentioned, but nothing very helpful about the cause and prophylaxis of such infections as summer diarrhœa of infants. There are some excellent tables of such things as incubation periods, but even these exhibit imperfections in the lack of any alphabetical or other definite arrangement. The illustrations are mostly borrowed and but fairly reproduced. The book was well conceived and well started, but it is, we venture to think, very far from being a complete success.

### CLINICAL PATHOLOGY.

Clinical Diagnosis. By Charles Phillips Emerson, A.B., M.D., late Resident Physician and Associate in Medicine, The Johns Hopkins University. Large 8vo. Pp. xxv. + 686; 5 Plates and 126 Figures. (Philadelphia and London: J. B. Lippincott Company. Second edition. 1908. Price 21s. net.)

The first edition of this work appeared in 1906. Its popularity is obvious when a second edition has been called for so soon. Nor is the volume before us merely a reprint of the other; fully one-half of the letterpress has been re-written, and there have been many alterations and additions, together with many fresh illustrations. The seven years' curriculum at the Johns Hopkins University permits of each student devoting a far longer time to clinical laboratory work than is possible in our five years' course. It would be next to impossible for every medical student in this country to have had personal experience of all the laboratory methods and tests dealt with in Dr. Emerson's volume. Many of them, indeed, might scarcely be required once in a lifetime by any single practitioner. From the simplest and commonest to the most complicated and rare medical laboratory tests, each is described fully, clearly, and well. It is on this very account, seeing that rare and common things receive almost equal attention, that the work is one that is essential to the libraries and clinical laboratories of hospitals, but is almost too big for the every-day use of practitioners. As a work of reference we think it admirable, though we object to its title. By "clinical" one usually understands "bedside," whereas the book, as its subsidiary or explanatory title tells us, deals with microscopy and chemistry in the laboratory. The only sense in which it merits the term "clinical" is this: that all the interpretations given to the laboratory results are essentially such as bear upon the clinical aspects of each case. It is a modernised von Jacksh. The work is excellent throughout, but we think that most practitioners would find one of the smaller books upon the subject both more convenient and less expensive.

Physiology and Pathology of the Urine. By J. Dixon Mann, M.D., F.R.C.P., Professor of Forensic Medicine in the University of Manchester. (London: C. Griffin and Co. 1908. Pp. xiv+324, with 30 figures. 8vo. Second edition. Price 10s. 6d. net.)

His well-known work upon forensic medicine makes the name of Dixon Mann so familiar to us all that we expect great things from him when he writes. We are bound to say that his monograph upon the urine fills us with disappointment. It would disappoint us whoever its author was; it disappoints us far more when its author is Professor Mann. The reason for our dissatisfaction lies partly in the fact that we are misled by the title of the volume. If the work were called "Methods for the Examination of Normal and Pathological Urines" we should expect it to contain little beyond the mere detailed accounts of how to test for and estimate substances that may be found in urine. But in the book before us that which bears the guise of a monograph upon the urine is little more than a compendium of chemical methods with only a few paragraphs and pages devoted to the urinary changes to be expected under different conditions of health and disease. The clinician will find far better books upon the subject, some costing less money and yet affording useful clinical and laboratory information not only upon urines, but also upon blood and so forth inside the same cover. Those whose work lies largely within a laboratory for pathological chemistry will no doubt find the book most useful as a compendium of methods of urinary analysis, ordinary and extraordinary; but from the point of view of the general practitioner or the consulting physician or surgeon we think the work fails in its professed object of serving "as a clinical guide in the diagnosis and treatment of disease."

### MISCELLANEOUS ITEMS.

Hints to Ships' Surgeons. By J. F. Elliott, L.R.C.S. (London: J. Bale, Sons, and Danielsson. 1908. Price 2s.)

If any evidence were needed as to the changed status of the ship's surgeon in late years, it is amply supplied by the increasing literature devoted to the duties of this class of practitioner. It is evident enough, when one comes to think of the dimensions of modern liners and of their enormous populations, that the position of medical officer thereon is one of vast responsibility and affords experience of the most varied kinds; and this entails with it the necessity for a high standard and improved status among the holders of this position. The advice of seniors on the many situations which test the abilities of those who thus go down to the sea in ships is particularly valuable because in so many instances the ship's surgeon is comparatively young. In the volume under discussion will be found a series of hints as to the conduct of the medical officer at sea in many of the vicissitudes which befall him. The treatise is perhaps less systematic than some that have already appeared on the subject; it does not aim at covering the ground completely, but it is reliable and practical as far as it goes. Anyone contemplating a first voyage in this capacity will be well advised to read it through.

First Lines in Dispensing. By E. W. Lucas, F.I.C., F.C.S. (London: J. and A. Churchill. 1908. 8vo. Pp. 166. Illustrated. Price 3s. 6d. net.)

Dispensing, as understood by the trained pharmacist, is an art which can only be mastered by practical experience, with the accompaniment of a comprehensive knowledge of pharmacy and allied subjects; but in cases where the operation of dispensing cannot be undertaken by persons who have undergone a pharmaceutical training it is desirable that those who take the place of the pharmacist should be able to fill the position in as capable a manner as their limited experience permits. While dispensing is an art which cannot wholly be learnt from books, such a volume as "First Lines in Dispensing" forms a useful substitute for a tutor and a serviceable guide to the student, as well as a practical reference-book for dispensers who are uncertain as to how a particular medicinal combination should be effected or how certain prescriptions should be manipulated. The book is introduced by some suggestions on the arrangement and fittings of a dispensary, and, after describing and explaining various processes which dispensing entails, the author treats of mixtures, powders, cachets, pills, suppositories, ointments, plasters, tablets, pastilles, and capsules, explaining general methods of procedure, furnishing typical examples, pointing out pitfalls, and giving useful hints. A few notes explanatory of the construction of prescriptions, a vocabulary of Latin terms, solubility tables, a list of synonyms, and a table of the doses of the more potent drugs are other features of the book. The author writes as a pharmaceutical chemist of long experience, and in every instance his advice can be relied upon; while as a past member of the Board of Examiners of the Pharmaceutical Society he has had opportunities of making himself acquainted with the direction in which students' difficulties most frequently lie.

From an Easy Chair. By Sir E. Ray Lankester, K.C.B., F.R.S. Pp. 144; paper covers. (London: Archibald Constable and Company, Limited. Price 1s. net.)

This is practically a reprint of a series of articles, which have appeared recently in the columns of the *Daily Telegraph.* The papers consist, to use the author's own words, of "brief notes in plain language on a variety of scientific matters." They are thus essentially designed to suit the popular palate, and the very varied information which they contain is served up in a pleasant and easily assimilable form. The title well describes the author's intention, and his volume of light scientific essays will no doubt prove of value and of interest to those for whom it is written. The last chapter, entitled "Cruelty Pain and Knowledge" deserves particular notice; for it deals in a temperate and broad-minded manner with the current misconception upon experiments on animals, and should prove of great assistance to those who are engaged in the defence of research.

Vital Economy: or, How to Conserve your Strength. By John H. Clarke, M.D. (London: T. Fisher Unwin. 1909. Price 1s. net.)

This book professes to have been written primarily for that class of persons who have just enough vitality and no more to enable them to get through their duties—those who are naturally delicate, those who are convalescing from acute illnesses, those who are "below par." It is clearly the writing of an elderly man, and one reads it in much the same way as one listens to the speech of age—with respect for the speaker but not always for what he says. The book is whimsical, and although there is much truth in some of the whimsies there is little enough in others. Some of the subjects dealt with are the following: the bath, and the dangers of routine baths; the advisability that certain patients should remain unwashed; the excesses to which ideas are carried in regard to fresh air, exercise, and stimulants; tea-drunkards; worriers, and the extravagance of worry; the varieties of holidays; and visitors to the sick. One cannot believe everything an author says when amongst his statements one reads that "a strongly magnetic person by taking the hand of another can give electric shocks which are plainly felt in the nerves and muscles of the arms of the second person"; and yet there are portions of the book, such as the opening paragraphs upon "Visiting the Sick," which will receive the fullest endorsement from every medical man.

Who's Who, 10s. net; Who's Who Year-Book, and The Writers' and Artists' Year-Book, 1909, 1s. each; and The Englishwoman's Year-Book and Directory, 2s. 6d. net. (London: A. and C. Black.)

These annual publications, and particularly the former two, have become almost indispensable to those men and women who take an active part in the work of the world, or who merely take an intelligent interest therein. The issues for 1909 will be found fully up-to-date and with various additions and improvements "Who's Who" now contains 23,000 biographies, every one of which has been submitted for personal revision; and its 2112 pages comprise a wonderful collection of compressed autobiographies. In the "Englishwoman's Year-Book" will be found much useful and accurate information about the medical profession as a career for women, together with a directory of medical women. "Who's Who Year-Book" is made up of tables which were formerly contained in the larger work, and comprises, among other useful tabulations, the names of the staffs of the great London hospitals.

---

"Londoner, M.D.," writes: In answer to the inquiry of "H. P." in your issue of December 12 as to a published account of Professor Schäfer's system of artificial respiration, it may be of interest to him to know that the Journal of the American Medical Association for September 5, 1908, contains such an account. Whether there is one published in England I cannot say, though it must be very probable. "H. P." will, however, find all he wants from the publication mentioned.

